# *Calanthe sieboldopsis* (Orchidaceae, Epidendroideae, Collabieae), a new species from Luoxiao Mountains, eastern China

**DOI:** 10.3897/phytokeys.145.49386

**Published:** 2020-04-10

**Authors:** Bo-Yun Yang, Huo-Lin Luo, Wei-Chang Huang, Dong-Jin Xiong, Shao-Lin Tan, Bo Li

**Affiliations:** 1 School of Life Sciences, Nanchang University, Nanchang, Jiangxi 330031, China; 2 Key Laboratory of Plant Resources in Jiangxi Province, Nanchang, Jiangxi 330031, China; 3 Shanghai Chenshan Botanic Garden, Songjiang District, Shanghai 201602, China; 4 Research Centre of Ecological Sciences, Jiangxi Agricultural University, Nanchang, Jiangxi 330045, China

**Keywords:** *Calanthe sieboldii*, Critically Endangered, Jiangxi Province, Jinggangshan Mountain, morphology

## Abstract

*Calanthe sieboldopsis*, a new species, is here described and illustrated from Luoxiao Mountains, Jiangxi Province, eastern China. It is morphologically similar to *C. sieboldii* Decne. ex Regel, but differs from the latter in having smaller flowers, longer spurs, rectangular mid-lobes with emarginate apex (*vs.* elliptic mid-lobes with mucronate apex), disc with 3 ridges and the proximal ends of the lateral 2 ridges enlarged with light reddish spots and minute white hairs (*vs.* disc with 5 ridges and 2 rows of white short hairs at base) and pollinia equal in size (*vs.* unequal in size). A preliminary risk-of-extinction assessment, according to the IUCN Red List Categories and Criteria, is given for the new species.

## Introduction

*Calanthe* R. Br. is the largest genus of the tribe Collabieae (in the subfamily Epidendroideae), according to the updated classification of the orchid family (Chase et al. 2015). The genus contains about 160 species of terrestrial orchids, commonly occurring in tropical, subtropical and temperate regions of Asia, Africa and the Pacific islands (Clayton and Cribb 2013), with a concentrated distribution of more than 50 species in China (Chen et al. 2009). Based on the latest infrageneric treatment, *Calanthe* was subdivided into five sections, viz., sect. Alpinocalanthe J.W.Zhai, Z.J.Liu et F.W.Xing, sect. Calanthe, sect. Ghiesbrechtia (A. Rich. & Galeotti) Schltr., sect. Puberula J.W.Zhai, Z.J.Liu et F.W.Xing and sect. Tricarinata J.W.Zhai, Z.J.Liu et F.W.Xing (Zhai et al. 2014). In the last two decades, new species of *Calanthe* have been discovered in China (Ren et al. 2011; Huang et al. 2015; Guo et al. 2017; Yu et al. 2017), Korea (Oh et al. 2015), Myanmar (Kurzweil 2013) and the Philippines (Naive 2017) and more discoveries can be expected, indicating that the diversity of *Calanthe* in Asia has not yet been fully revealed.

**Figure 1.**
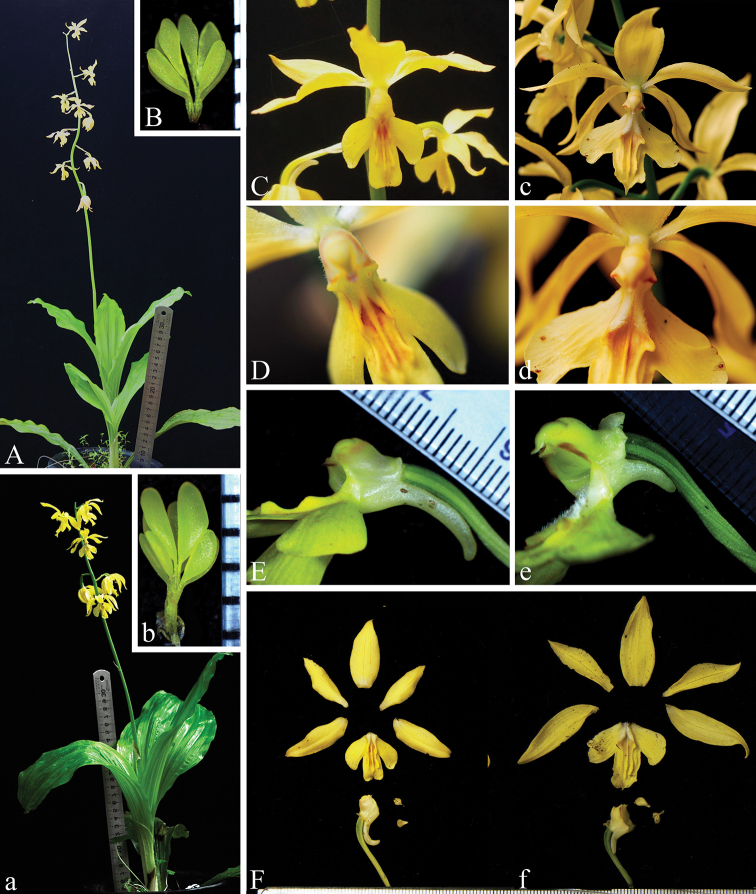
Morphological comparison between *Calanthe sieboldopsis* B.Y.Yang & Bo Li, sp. nov. (**A–F**) and *C. sieboldii* Decne. ex Regel (**a–f**) **A, a** habit **B, b** pollinia **C, c** flowers **D, d** column (top view) and base of lips **E, e** column (lateral view) and spur **F, f** dissection of a flower.

In order to ascertain the orchid diversity in Jiangxi Province of eastern China for preparing the Orchidaceae account of *Flora of Jiangxi*, we have carried out continuous field surveys from 2008 to 2019. When identifying specimens collected from Jinggangshan National Nature Reserve in 2017, we encountered an unknown collection of *Calanthe*, which is superficially similar to *C. sieboldii* Decne. ex Regel, but differs in having smaller flowers, longer spurs and emarginate lip mid-lobes with different characteristics of the disc (Figure 1). After carefully comparing the plant with all known congeneric taxa and consulting the relevant literature, we confirm that the plant represents an undescribed new species which is described here.

## Methods

The locality of the new species in Jinggangshan National Nature Reserve of Jiangxi Province was revisited in April 2018 and one flowering individual was collected for the line-drawing. Expecting to discover more populations of the new species, we expanded our field surveys from Jinggangshan Mountain to the whole mountain range, Luoxiao Mountains, in 2018 and 2019 and finally found an additional population on Xianyueshan Mountain of Hunan Province. The morphological description is based on flowering plants in the two populations and morphological measurements were taken using a ruler and a Vernier caliper from at least 10 individuals. Flowers were dissected and photographed under a stereo dissecting microscope (StereoZoom Leica S8 APO, Leica Microsystems 2017).

Herbarium specimens of the genus *Calanthe* were examined at IBK, IBSC, JIU, JXU, KUN, LBG, NAS, PE and SYS (for herbarium acronyms, see Index Herbariorum: http://sweetgum.nybg.org/ih). Fresh plants of *C. sieboldii*, the most similar species of the new species, were collected from Tianmashan Mountain of Jiangxi Province in 2019 for morphological comparisons. The distribution map was prepared using geo-referenced locations, data obtained from herbarium specimens and our own field observations. The conservation status of the new species was evaluated, based on the guidelines of the International Union for Conservation of Nature (IUCN 2019).

## Taxonomy

### Calanthe sieboldopsis

Taxon classificationPlantaeAsparagalesOrchidaceae

B.Y.Yang & Bo Li sp. nov.

4D94B8E1-63A9-5F09-8946-DD4AF4A4FEC2

urn:lsid:ipni.org:names:77209332-1

**Figure 2.**
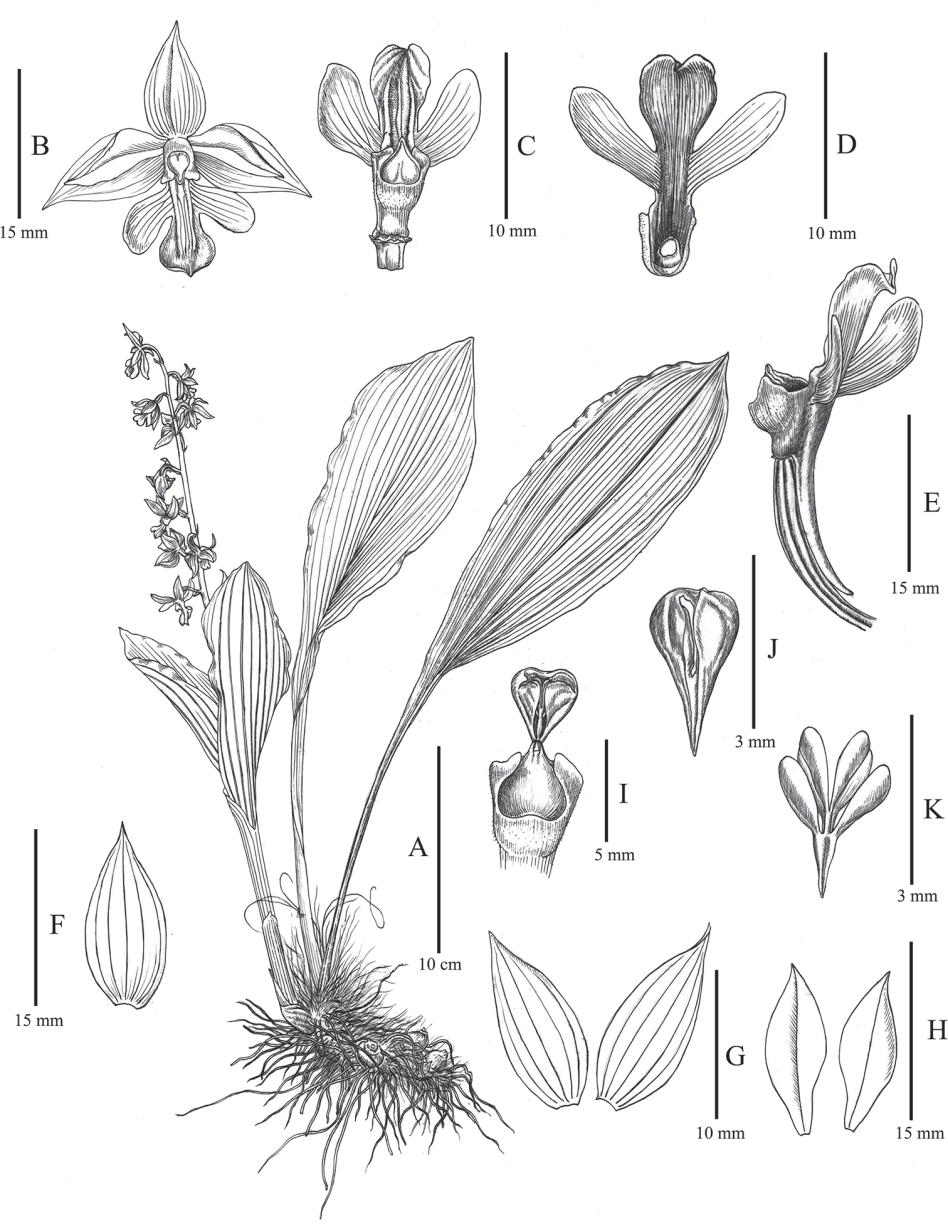
Line drawing illustration of *Calanthe sieboldopsis* B.Y.Yang & Bo Li, sp. nov. **A** habit **B** flower **C** column and lip (top view) **D** lip (bottom view) **E** ovary, column, lip and spur (lateral view) **F** dorsal sepal **G** lateral sepals **H** petals **I** column, rostellum and anther cap (uncovered) **J** anther cap (top view) **K** pollinia.

异大黄花虾脊兰 Figures 1A–F , 2

#### Diagnosis.

This species is most similar to *Calanthe sieboldii* in habit, gross morphology and flower colour, but differs from the latter in having smaller flowers (dorsal sepal 15–20 mm in length in *C. sieboldopsis vs.* 22–30 mm in *C. sieboldii*, petals 15–17 in length *vs.* 19–24 mm), longer spurs (12–14 mm *vs.* 6–8 mm), lip mid-lobe nearly rectangular with emarginate apex (*vs.* elliptic with mucronate apex), disc with 3 ridges and the proximal ends of the lateral 2 ridges enlarged with light reddish spots and minute white hairs (*vs.* disc with 5 ridges and 2 rows of white short hairs at base), pollinia equal in size (*vs.* unequal in size with the lower 4 smaller and the upper 4 larger).

#### Type.

China. Jiangxi Province: Ji’an City, Jinggangshan Mountain, Jinggangshan National Nature Reserve, in the valley, near margins of evergreen broad-leaf forest, 26°25'17"N, 114°35'02"E, ca. 540 m a.s.l., 19 April 2017, *B.Y.Yang 095* (Holotype: CSH!; Isotypes: JXU!).

#### Description.

Plants 35–45 cm tall. Rhizome elongate, thick. Pseudobulbs small, inconspicuous, obscured by the bases of the leaves, with 3–5 basal sheaths. Leaves 4–7, well developed and spreading at anthesis, not deciduous; blade broadly elliptic, 14–28 × 6.5–9.0 cm, apex acute; petiole-like base 9.5–16 cm long, usually forming a pseudostem, 8.5–10 cm long, 1.0–1.5 cm in diam. Scape arising from leaf axil, 30–45 cm tall, sparsely puberulent; rachis 15–25 cm long, laxly 8–11-flowered; floral bracts persistent, lanceolate, 5.0–8.5 mm long, puberulent, apex acuminate. Flowers bright yellow, large, slightly fleshy, glabrous except for lip base; pedicel and ovary 18–20 mm long, minutely puberulent. Dorsal sepal elliptic, 15–20 × 5.0–6.5 mm, apex acute; lateral sepals narrowly oblong, 12–15 × 4–6 mm, apex acute. Petals obovate-lanceolate, 15–17 × 4.5–5.5 mm, base narrowed, apex acute; lip adnate to entire length of column wings, spreading horizontally, yellow, mottled red at base, deeply 3-lobed; lateral lobes oblong-elliptic or falcate-obovate, oblique, 6.5–8.0 × 4.5–5.5 mm, apex obtuse-rounded; mid-lobe nearly rectangular, 6.0–8.5 × 4.5–5.5 mm, apex emarginate; disc with 3 ridges, extending to middle of mid-lobe; the proximal ends of the lateral 2 ridges enlarged, with light reddish spots and minute white hairs; spur curved, cylindrical, 12–14 mm, inside puberulent. Column 5.0–6.0 mm, thick, wings decurrent and connecting to ridges on disc; rostellum 2-lobed; anther cap beaked; pollinia 8, clavate, equal in size, grouped into two clusters, each with a short caudicle, 0.5 mm long, attached to an elliptic viscidium.

#### Phenology.

Flowering was observed from early April to early May and fruiting from late April to early June.

#### Distribution.

**Figure 3.**
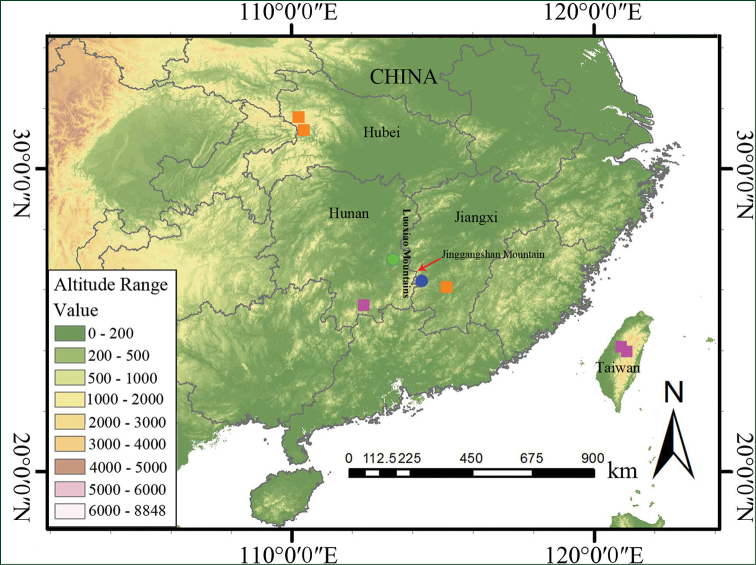
Distribution map of *Calanthe sieboldopsis* (blue and green circles show the type locality and another occurrence, respectively) and *C. sieboldii* (purple and orange squares show historical records and the recently-discovered localities, respectively).

The species is currently known only from two sites: one is the type locality in Jinggangshan National Nature Reserve, Jinggangshan Mountain, Luoxiao Mountains of Jiangxi Province and another in Xianyueshan Mountain of Liling City, Hunan Province (Figure 3). The two localities belong to the same large mountain range, Luoxiao Mountains, which are the provincial border of Jiangxi and Hunan provinces. Plants of both populations grow in wet valleys and near margins of evergreen broad-leaf forest at elevations of 400–600 m a.s.l.

#### Etymology.

The specific epithet “*sieboldopsis*” is a combination of two phrases “*siebold*” (which is derived from the specific epithet of the species *C. sieboldii*) and “*opsis*” (which means resembling), indicating that *C. sieboldopsis* is most similar to *C. sieboldii*.

#### Preliminary conservation assessment.

The species is only known from two separate localities in the Luoxiao Mountains range: one is in the Jinggangshan National Nature Reserve of Jiangxi Province, where its habitat is well protected and 21 mature individuals were counted; the second locality in Xianyueshan Mountain of Liling City, Hunan Province, is not located in a nature reserve and only five flowering individuals have been found. Based on our observations, deforestation is the main threat to the Hunan population. Though there are probably other populations that have not been discovered in the Luoxiao Mountains or adjacent regions, we have carried out thorough field surveys in Jiangxi Province for more than 10 years and, so far, found that the total mature individuals of *C. sieboldopsis* are much less than 50, thus the new species should be categorised as critically endangered (CR) under criteria D, following the *Guidelines for Using the IUCN Red List Categories and Criteria* (IUCN 2019).

#### Other specimens examined.

*Calanthe sieboldopsis*: China. Hunan Province: Liling City, Xianyueshan Mountain, in the valley, 27°38'02"N, 113°27'22"E, 410 m a.s.l., 11 April 2018, *B.Y.Yang 119* (JXU!); Jiangxi Province: Ji’an City, Jinggangshan National Nature Reserve, 26°25'17"N, 114°35'02"E, 540 m a.s.l., 25 April 2018, *B.Y.Yang 122* (JXU!); *ditto*, 10 April 2019, *B.Y.Yang 151* (JXU!).

*Calanthe sieboldii*: China. Hubei Province: Shennongjia Forestry District, Shennongjia National Nature Reserve, roadside, 31°23'51"N, 110°34'55"E, 475 m a.s.l., 11 August 2008, *D.G.Zhang zdg7268* (JIU!); Shennongjia Forestry District, Yangri Village, mountain slope, in evergreen broad-leaf forests, ca. 2000 m a.s.l., 15 May 2012, *D.G.Zhang zdg6189* (JIU!); Hunan Province: Linwu County, Xishan National Forest Park, in evergreen broad-leaf forests, 570 m a.s.l., 4 April 2014, *X.L.Yu et al. 140404* (six sheets, CSFI!); Jiangxi Province: Ji’an City, Qingyuan District, Tianmashan Mountain, in forests, 26°40'54"N, 115°24'19"E, 560 m a.s.l., 15 April 2015, *B.Y.Yang JA201504017* (JXU!); *ditto*, 22 April 2019, *B.Y.Yang 154* (JXU!); Taiwan Province: Xinzhu County, Jianshi Town, along Shihlu Historic Road, near Paishih Drawbridge, on steep rocky slope, 24°33'18"N, 121°13'38"E, 1420 m a.s.l., 21 February 2002, *Y.Y.Huang 979* (HAST!); *ditto*, Jianshi Town, Litungshan Mountain, way to Nalo-Yulao, 7 March 1987, *H.J.Su 7772* (HAST!).

#### Note.

When preparing the Orchidaceae account for *Flora of Jiangxi*, we have carried out many years of field surveys, resulting in the discovery of a large number of new records for Jiangxi and a few taxa new to science (e.g. Yang et al. 2017, Wang et al. 2018). In *Calanthe*, 11 species have been found in Jiangxi Province, including the new species *C. sieboldopsis* and the newly-recorded *C. sieboldii* (Wang et al. 2018). Though in the latest revision of the genus *Calanthe*, *C. sieboldii* was reduced as a synonym of *C. striata* Lindl. (Clayton and Cribb 2013), this taxonomic treatment was not widely accepted in recent literature (e.g. Zhai et al. 2014; Zhou et al. 2016; Jin et al. 2019), thus we here recognise *C. sieboldii* as a separate species. *Calanthe sieboldii* was previously mainly reported from Ryukyu Islands of Japan, from Korea and from Hunan and Taiwan provinces of China (Chen et al. 2009), but it was recently discovered from Shennongjia National Nature Reserve of Hubei Province (Xie et al. 2017) and Ji’an City of Jiangxi Province (Wang et al. 2018) (Figure 3). *Calanthe sieboldopsis* is superficially most similar to *C. sieboldii* because both of them are characterised by having large bright yellow flowers, but they are clearly different from each other in flower size, spur length, shape and characteristics of mid-lobes of lips and pollinia size (Figure 1). Amongst these differences, the shape of the lip mid-lobe and the characteristics of the disc could be used as the main identification trait to distinguish the two species.

### Key to the species of *Calanthe* in Jiangxi Province of China

**Table:** 

1	Floral bracts caducous; rostellum unlobed	***C. clavata***
–	Floral bracts persistent; rostellum 2- or 3-lobed	**2**
2	Lip spurless	**3**
–	Lip spurred	**4**
3	Sepals to 7 mm long; sepals and petals not reflexed	***C. tsoongiana***
–	Sepals 15–20 mm long; sepals and petals strongly reflexed	***C. reflexa***
4	Lip adorned with wart-like calli on disc	**5**
–	Lip adorned with ridges or lamellae	**7**
5	Mid-lobe of lip entire or shallowly 2-lobed	***C. sylvatica***
–	Mid-lobe of lip divided by a deep sinus into 2 lobules	**6**
6	Leaves with several silver-grey bands on adaxial surface; spur 15–19 mm long; flowers yellowish-green	***C. argenteostriata***
–	Leaves without silver-grey bands; spur ca. 10 mm long; flowers white, sometimes tinged purplish-violet or occasionally purplish-red	***C. alismifolia***
7	Disc with 3 membranous, triangular lamellae	***C. discolor***
–	Disc with 3–5 parallel ridges	**8**
8	Sepals and petals yellowish-brown, white or pink, occasionally flushed purple	**9**
_	Sepals and petals bright yellow	**10**
9	Sepals and petals white or pink; disc without brown spots; spur 20–32 mm long	***C. aristulifera***
–	Sepals and petals yellowish-brown; disc with 4 brown spots; spur 10–18 mm long	***C. graciliflora***
10	Lip mid-lobe elliptic with mucronate apex; spur 6–8 mm long; pollinia unequal in size	***C. sieboldii***
–	Lip mid-lobe nearly rectangular with emarginate apex; spur 12–14 mm long; pollinia equal in size	***C. sieboldopsis***

## Supplementary Material

XML Treatment for Calanthe sieboldopsis
